# Research of Wafer Level Bonding Process Based on Cu–Sn Eutectic

**DOI:** 10.3390/mi11090789

**Published:** 2020-08-20

**Authors:** Daowei Wu, Wenchao Tian, Chuqiao Wang, Ruixia Huo, Yongkun Wang

**Affiliations:** 1 School of Electro-Mechanical Engineering, Xidian University, Xi’an 710000, Shanxi, China; dwwu@stu.xidian.edu.cn (D.W.); wangchuqiao@xidian.edu.cn (C.W.); ykwang@xidian.edu.cn (Y.W.); 2 Xi’an Institute of Microelectronic Technology, Xi’an 710000, Shanxi, China; wchuqiao1611@163.com

**Keywords:** Cu–Sn bumps, wafer-level eutectic bonding, intermetallic compounds

## Abstract

In 3D-system packaging technologies, eutectic bonding is the key technology of multilayer chip stacking and vertical interconnection. Optimized from the aspects of the thickness of the electroplated metal layer, the pretreatment of the wafer surface removes the oxide layer, the mutual alignment between the wafers, the temperature of the wafer bonding, the uniformity of pressure and the deviation of the bonding process. Under the pretreatment conditions of plasma treatment and citric acid cleaning, no oxide layer was obtained on the metal surface. Cu/Sn bumps bonded under the condition of 0.135 Mpa, temperature of 280 °C, Sn thickness of 3–4 μm and a Cu-thickness of five micrometers. Bonded push crystal strength ≥18 kg/cm^2^, the average contact resistance of the bonding interface is about 3.35 mΩ, and the bonding yield is 100%. All performance indicators meet and exceed the industry standards.

## 1. Introduction

The three-dimensional integration technology based on through-silicon via (TSV) achieves homogeneous or heterogeneous three-dimensional integration by making vertical via, which can greatly reduce the size of the chip, while improving interconnection density and electrical performance [1,2]. This technology has broad application prospects in network big data and memory manufacturing, as well as MEMS systems and has become the fourth-generation advanced packaging technology with the most development potential [3]. However, the impact of packaging on reliability is inevitable. Among them, bonding technology is one of the key technologies for 3D integration and packaging. Bump and bumpless interconnections are two mainstream interconnection methods [4,5]. Bumpless interconnection refers to the direct bonding between the upper and lower structures including the interconnection layer and the insulating layer, which is generally used for structures with a bonding pitch of less than 10 μm. Bump interconnection refers to making bumps on the interconnect layer and bonding to realize the interconnection of the upper and lower chips. Therefore, the reliability problem caused by the ultra-fine pitch microbump interconnection will become more severe [6].

The currently achievable 3D-bonding schemes include metal-to-metal direct bonding and solder bonding, etc [7,8]. Metal–metal direct bonding is mainly based on the interdiffusion of atoms between two bonding surfaces under high temperature and high pressure, such as Au–Au, Cu–Cu, and the bonding time is long [9,10]. This process condition will cause severe thermal shock to pre-embedded devices in the through–via process after TSV and may fail. In addition, the high temperature will cause greater thermal stress in the internal TSV structure, which is prone to deformation and fracture. Device reliability, device manufacturing yield, and technology applicability are all under great pressures [11]. Solder bonding realizes the bonding by heating, melting, reflowing and infiltrating low melting point metals or metal alloys, such as Sn, In–Sn, which avoids the negative effects of high temperature processes, but the process is easy to melt back, and the bonding process is reversible, which also cannot be applied to TSV stereo integration [12].

To this end, this paper studies high-density Cu–Sn microbump eutectic bonding process. Microbumps were produced by electroplating on 8-inch silicon wafers. The thickness of bonding materials, structure and layout were designed and optimized. By comparing different prebonding pretreatment and bonding processed, intermetallic compounds at the interface with stable performance were obtained, which was Cu_3_Sn. The bonding realized the low resistance electrical performance connection of the device and obtains higher reliability, and it could be applied to all wafer-level integration of multilayer devices. It not only realizes large-size wafer-level bonding, but also meets mass production requirements. The bonding process was optimized to meet the strict oxidation requirements of CuSn. Integrating pretreatment and bonding, it achieves high yield. The testing and evaluation methods had also been improved, and related methods were patented.

## 2. Experimental Method

In this paper, the bonding material system, bonding layer structure, wafer lithography pattern was designed, and the bonding process was optimized to realize large-scale wafer-level Cu–Sn eutectic bonding.

### 2.1. Layout Design

In order to realize the electrical performance test of the bonding products, the purpose was to used a series of bumps designed with a “daisy chain” with pad, by monitoring the size of the contact resistance, to comprehensively, accurately, effectively, real-time monitor and reflect the bonding process Bonding effect. Not only could the yield of the entire bonding surface be monitored in a timely and effective manner, but the wafer failure problem in the subsequent process due to poor bonding could be predicted or avoided in advance [13,14].

**Figure 1:**
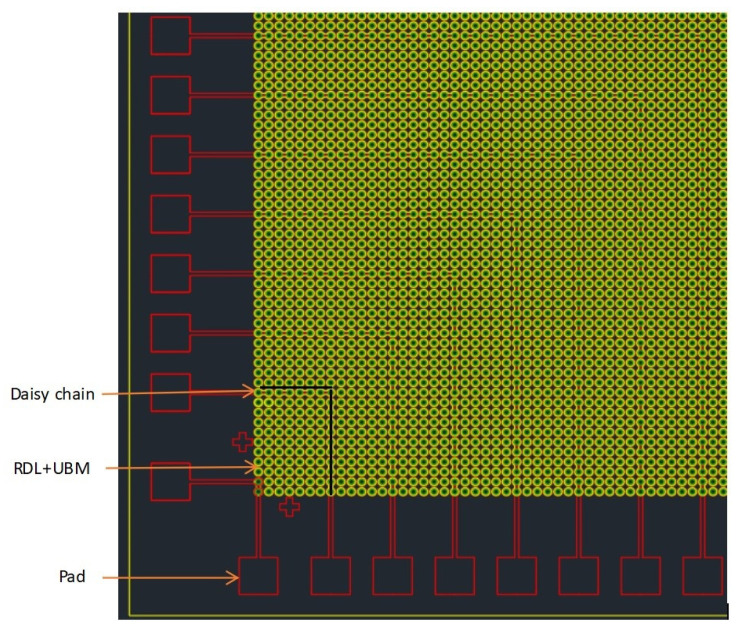
Wafer with pad, bumps and daisy chain partial layout (quarter of die, the green bumps in figure are the superposition of redistribution layer (RDL) and Under Ball Metal (UBM).

Specific design ideas: design different bonding patterns for the upper and lower 8-inch Si wafers, respectively. One side was a series bump structure with pad and “daisy chain”, which was the upper bonding surface of the wafer-level bonding. Its pad was for testing resistance and used for current conduction. After the bumps were each N independent bumps on the horizontal axis and the vertical axis, a series of “fishbone” series bumps were added, that was—each two two-two series—forming a test for bonding. The conductive lines of the intact bonding interface were formed, and this design was distributed to each die of the entire wafer. Figure 1 was a partial area of this layout that was, the layout of the lower left part, and the graphic distribution of the remaining upper left part, lower right part and upper right part was the same.

**Figure 2:**
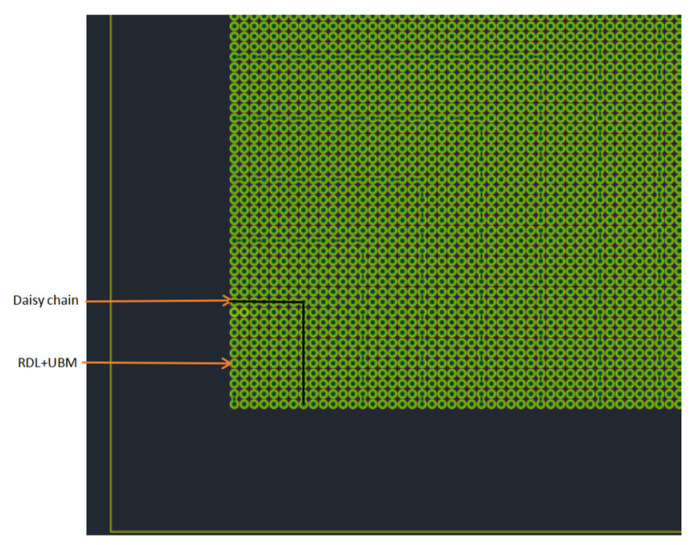
Wafer with only bumps and daisy chain partial layout (quarter of the die, the green bumps in figure are the superposition of RDL and UBM).

The other side was a tandem bump structure with only “daisy chain”, which was the lower bonding surface of wafer-level bonding, and its “daisy chain” bumps were each N independent bumps on the horizontal and vertical axes. After the point, add a series of “fishbone” series bumps, that was, two two-two strings in series, forming a conductive path line that could be used to test whether a good bonding interface was formed after bonding, and this design was distributed in the whole die for each wafer. Figure 2 shows the wafer with only bumps and the lower left part of the daisy chain layout, and the remaining upper left part, lower right part and upper right part had the same pattern distribution.

**Figure 3:**
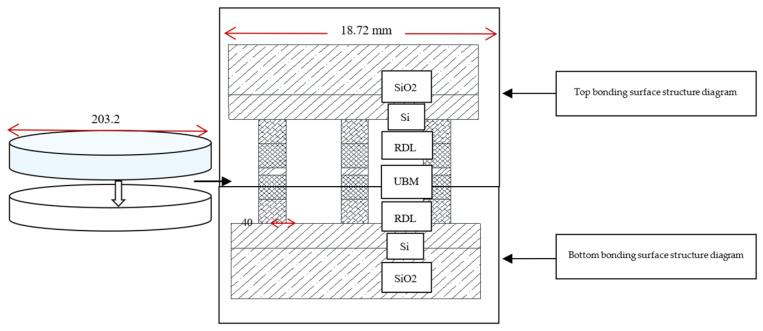
Schematic diagram of the structure after bonding.

The specific layout size designed in this experiment, in which the bump size was designed as a circular bump with a diameter of 40 μm (the bonding experiment was also applicable to other sizes, this article chose the bump size to be 40 μm) and a pitch of 20 μm (“pitch” means the distance from the edge of the bump to the edge of the bump, the distance from the center of the bump to the center of the bump was 60 μm), every 6 micro bumps had a series of bumps with a daisy chain as a circuit conduction and each surface includes 92 dies. The area of each die was 18.72 mm ∗ 18.72 mm. Each die contains 44,100 bumps, including 3178 on the daisy chain. After the production of the two bonding surfaces was completed, the two wafer surfaces were bonded and the structure diagram after bonding was shown in Figure 3. After bonding, the bumps were all columnar and the bonding surfaces were all horizontal.

### 2.2. Sample Production

First, we prepared two 8 inch, 700 μm Si wafers as top and bottom to be bonded and deposit 1-μm-thick SiO_2_ as an insulating layer on the surface; Then, physical vapor deposition (PVD) was used to sputter redistribution layer (RDL) that TiCu was 0.1/0.4 μm, where Ti serves as an adhesion layer and a barrier layer, Cu served as an electroplating seed layer. Then photolithography was used for the opening area of the microbump, and the thickness of Cu was increased to 4 μm by electroplating. After that, the bumps were patterned, and the metal of the bonding layer was plated. Among them, the electroplated Cu and Sn were compared with samples of different thicknesses. After the plating, the seed layer other than the bumps was peeled off, and the two wafers to be bonded were completed [15].

#### 2.2.1. Wafer Surface Cleaning

First, a plasma cleaning machine with an O_2_ flow rate of 90 sccm, an N_2_ flow rate of 10 sccm, and a power of 300 W was used to perform plasma cleaning 360S on bare wafers. Then, we used a wafer surface cleaning machine with megasonic cavity parameters: frequency 1 MHz, power 55 W, megasonic cleaning speed 50 rpm, time 120 S for megasonic cavity cleaning. Then we used 2000-rpm speed, time 120 S for high-speed spin-drying; then used iso-propyl alcohol (IPA) Rinse for 120 S to further remove moisture. The purpose of plasma cleaning, megasonic cleaning and IPA cleaning was to clean the particles and impurities on the surface of the wafer while taking away the water on the surface of the wafer. Finally, we used a N_2_ oven for further baking at 125 °C for 30 min to ensure the cleanliness, drying and subsequent bonding of the wafer surface.

#### 2.2.2. SiO_2_ Deposition on the Wafer Surface

**Figure 4:**
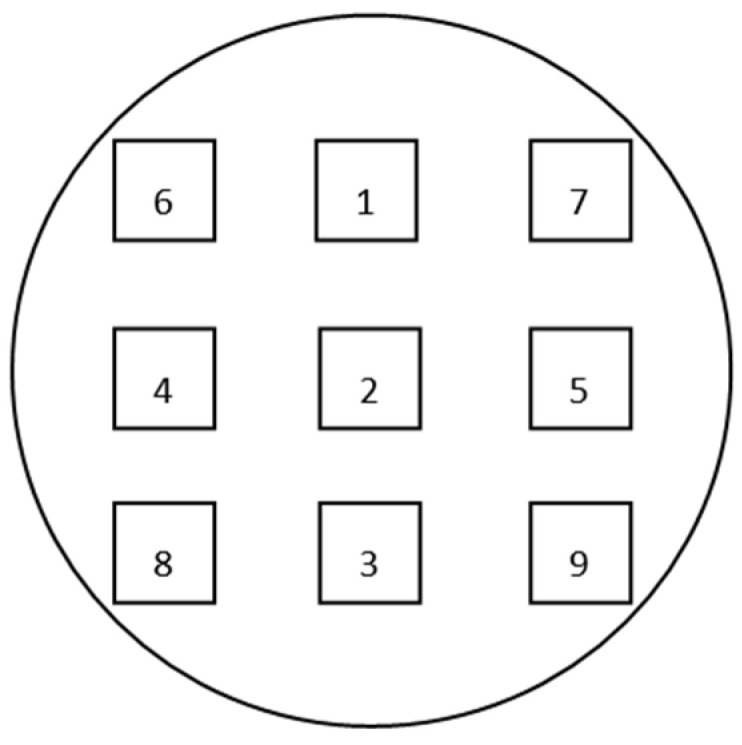
Schematic diagram of the location of the film thickness test point.

A plasma chemical vapor deposition method was used to deposit a 1-μm SiO_2_ thin film on the top surface Si wafer surface and the bottom surface Si wafer surface. The thickness could be measured by a nonmetal film thickness measuring instrument. If the SiO_2_ layer was too thick, it may affect the subsequent process and cause the reliability of the bump to decrease. The test method was a nine-zone nine-point test method, See Figure 4. We calculated the uniformity according to the results of the nine-zone test, which requires <2%.

#### 2.2.3. Wafer RDL TiCu Sputtering

The physical vapor deposition method was used to deposit TiCu on the top surface Si wafer surface and bottom surface Si wafer surface with SiO_2_ film deposited, which requires Ti 0.1 μm and Cu 0.4 μm. Among them, Ti acts as an adhesion layer and barrier layer, and Cu acts as an electroplating seed layer. Bump electroplating was performed in combination with photolithography and electroplating methods. After the deposition was completed, we used a stress tester to test the wafer surface warp Curvature test. After the film was deposited, as the temperature decreased, due to the difference between the thermal expansion coefficient of the substrate and the film, resulting in stress and bending. Wafer warpage test method was as follows: we selected two vertical inspection lines that cross the center of the wafer and selected 3 points for each inspection line [16].

#### 2.2.4. RDL Graphic

In order to ensure the cleanliness of the wafer surface, IPA was used for rotary surface cleaning. Then, the surface of the wafer was coated with 4620 photoresist to 10 μm. The role of photoresist was to engrave RDL patterns on the surface of the material. After the coating was applied, it was exposed and then developed and baked. We made sure there were no bad phenomena such as unclean development, glue pouring and residual glue after the patterning; the opening size was controlled at 60 ± 5 μm.

#### 2.2.5. RDL Preparation

Before electroplating, the residual glue on the wafer surface was removed, and the dry glue remover was used to etch using the combination of O_2_ and Ar. A 5-μm-thick Cu layer was electroplated by electroplating process, and DC voltage was used for electroplating. In the case of the same electroplating solution, the most important process parameters that affect the effect of Cu electroplating were current density and waveform. Larger current density could shorten the plating time, but due to excessive current density will lead to poor plating uniformity, rough metal layer and other problems. By optimizing the time distribution of the current density and the concentration of each additive in the potion, the compactness and uniformity of the plating could be ensured. After the plating was completed, the thickness of the plating was tested by X-ray fluorescence spectrometer (XRF). The test method was the nine-zone nine-point test method, as shown in Figure 4. After the test was completed, the wet photoresist was removed [17]. The current density of different parts of the electroplating process and the brands of Cu and SnAg plating solutions were shown in Table 1.

#### 2.2.6. Graphic Bumping

In order to ensure that there was no residual glue on the surface of the wafer, a dry glue remover was used to etch using a combination of O_2_ and Ar. Then apply wafer surface glue, used THB111 photoresist to apply glue 10 μm, apply glue and then expose, then develop and bake. After patterning, ensure that there were no abnormalities such as unclean development, glue pouring and residual glue; the opening size was 40 ± 5 μm.

#### 2.2.7. Bump Plating

**Figure 5:**
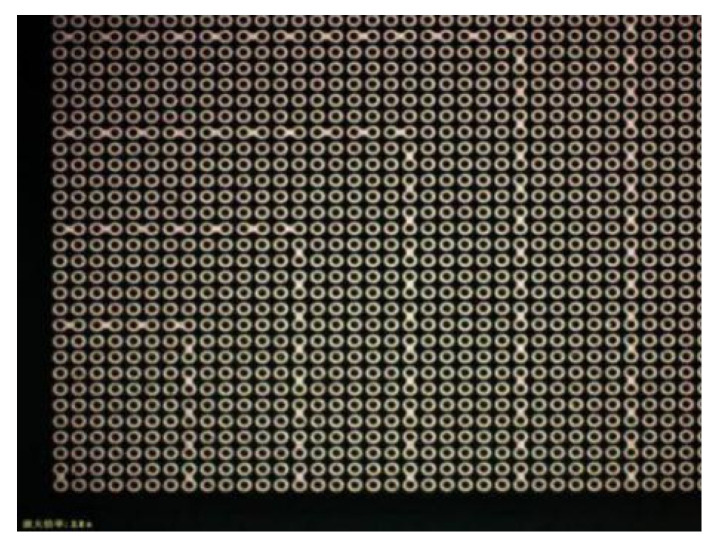
Partial array of wafers with only bumps and daisy chains.

**Figure 6:**
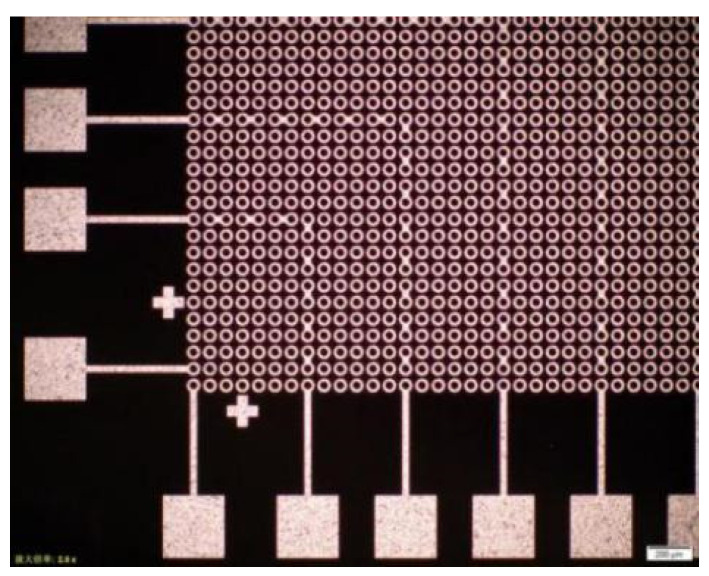
Partial array of wafers with pads, bumps and daisy chains.

**Figure 7:**
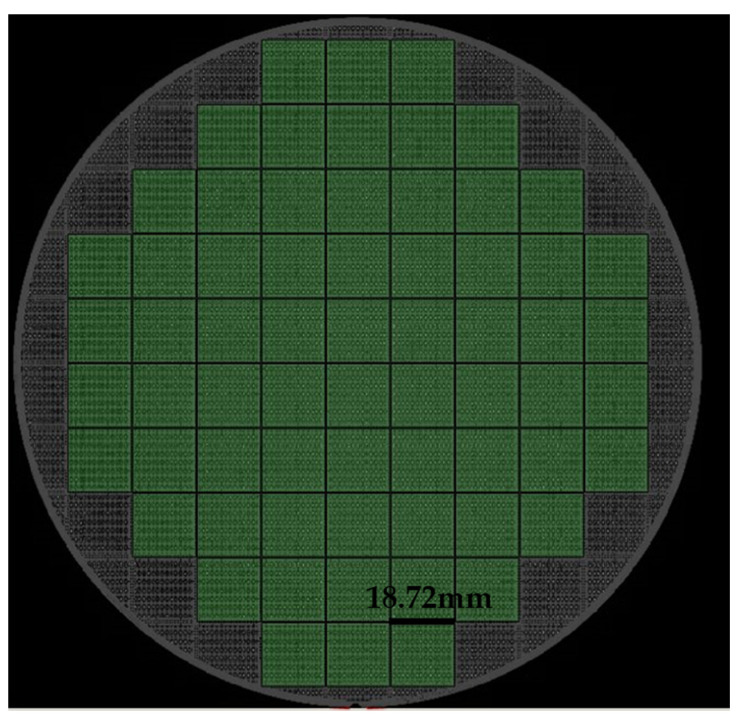
Distribution of the whole die scanned by automated optical inspection (AOI).

**Figure 8:**
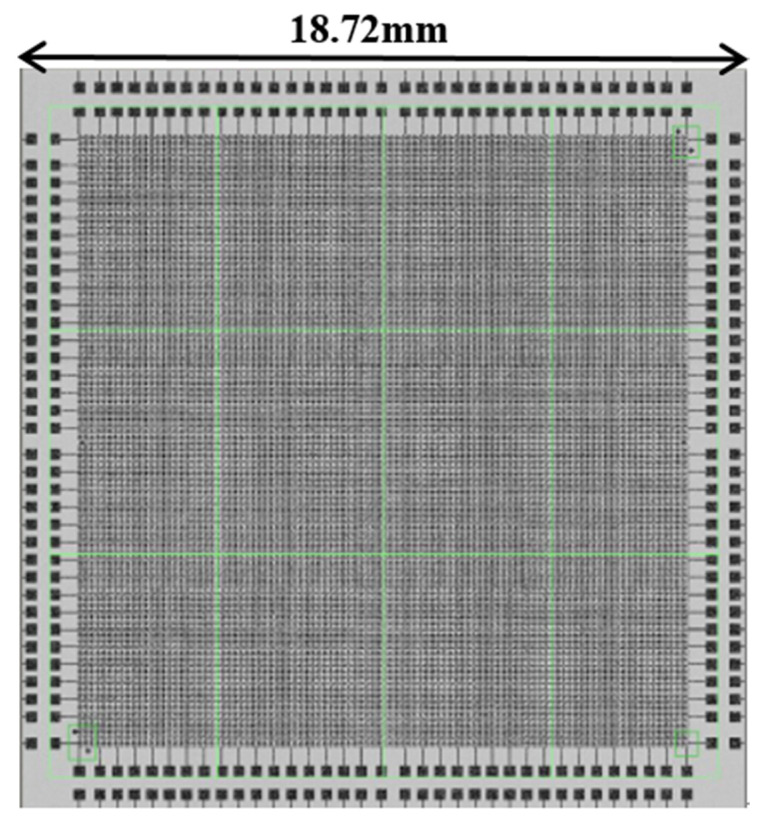
Morphology of a single die scanned by AOI.

After the bump patterning was completed, the wafer electroplating machine was used for bump plating. The plating thickness requirements were: Cu 5 μm for the top bonding surface; Cu 5 μm for the bottom bonding surface and SnAg 3 μm (The ratio of SnAg plating was 100:1). A small amount of Ag was selected here to improve the stability, corrosion resistance and conductivity of Sn during electroplating. After the plating was completed, the thickness of the plating was tested by XRF. The test method was the nine-zone nine-point test method [18], as shown in Figure 4. After the test was completed, the wet photoresist was removed. Figure 5 is a single-die picture after the top bonding surface bumps were made. Figure 6 is a single-die picture after the bottom bonding surface bumps were finished. The optical inspection equipment was used to detect the high consistency of the bumps on the wafer surface, and the MAP was formed as shown in Figure 7 and Figure 8. The edge of Figure 7 was an invalid die, showing gray. Except for the invalid die, the whole piece was uniform and highly consistent. The yield could reach 100%. The distribution of die on the top and bottom surfaces was the same. Figure 8 shows the morphology of the scanned die top and bottom. When bonding, the bumps on the top and bottom surfaces correspond to each other.

### 2.3. Bonding Process

After the bumps on the two bonding surfaces were completed, the Cu/Sn bump-bonding process was performed. The process of Cu/Sn bonding mainly included 4 steps [19]:

- Sample pretreatment before bonding:
  1. Plasma cleaning;
  2. After washing with citric acid (concentration: 1:10 = citric acid: water), rinse and dry with IPA;
  3. Oven drying; in order to obtain the optimal pretreatment process parameters, different pretreatment process conditions are compared, parameter settings are shown in Table 2.
- Sample alignment:
  After surface treatment, the wafer alignment machine is used to align the upper and lower wafers. According to the upper and lower pairs of CCD (charge coupled device) camera of the wafer alignment machine, the lower CCD looks at the upper wafer and the marks of the upper and lower wafers are found and aligned.
- Wafer-level bonding:
  In order to obtain the optimal bonding parameters, different bonding temperatures, pressures, temperature holding time and pressure holding time are compared, Table 3 are the settings of parameter. Too much temperature rise rate will cause voids in the bonding interface.

## 3. Results and Discussions

In the cleaning pretreatment method before bonding, the main gases for plasma cleaning were Ar, N_2_, H_2_, the purpose was to remove surface oxides and residual impurities; after the citric acid reacts with the surface oxide layer, then used IPA to clean and spin dry, in order to Take away the residual liquid and water on the bonding surface; Group 4 was the best pretreatment process setting. The non-baking was to prevent the bonding surface from oxidizing during the oven baking process. This proves that the segmented citric acid cleaning was more effective in removing the oxide layer, and the IPA cleaning was followed by oven baking. Since the oven was not a reducing atmosphere, it was likely to cause secondary oxidation, so it was better not to perform baking. Therefore, we choose Group 4 as the best process.

**Figure 9:**
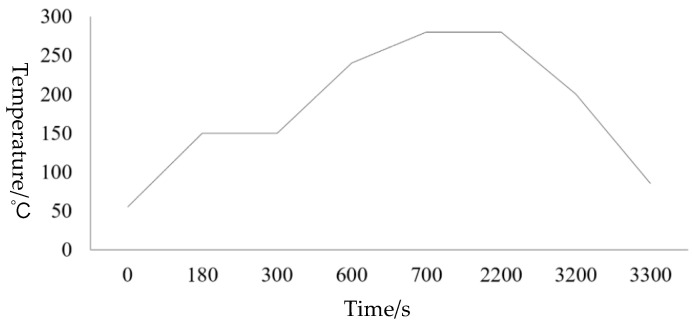
Curves of bonding temperature.

Among the wafer-level bonding methods with different parameters, Group 3 was the optimized combination of wafer-level bonding parameters. Figure 9 is a graph of the bonding temperature curve in which the optimized bonding parameters were reflected in the bonding process. (1) During the bonding process, in order to effectively prevent oxidation, the reducing mixed gases N_2_ and H_2_ were introduced; at the same time, the temperature was first raised to 150 °C for pretreatment to remove moisture from the sample surface. In order to make up for the cleaning pretreatment without baking, we directly moved this step to the bonding machine to prevent the oxidation of the baking process of the oven; (2) Then, we raised the temperature to 240 °C, pressure 7000-N and prebonded for 5 min; Increase the prebonding step to make Sn first enter the molten state for prepressing, so as to allow Cu/Sn to diffuse more fully; (3) After that, raise the temperature to 280 °C with the constant pressure. Cu–Sn completely infiltrated and forms an intermetallic compound and then complete bonding [20]; The reaction was carried out at different times during this process. When the reaction took place for 25 min, the complete stable product Cu_3_Sn were formed and at 20 min and 15 min, two compounds will appear at the bonding interface, namely Cu_3_Sn and Cu_6_Sn_5_. The reason was that Cu_6_Sn_5_ did not had enough time to continue to react with Cu to form Cu_3_Sn [21]; (4) Cooling: in order to reduce thermal stress due to the different thermal expansion coefficient between Si wafer and different metals, slowly lowering the temperature to 150 °C at a lower rate, then evacuate and fill N_2_ to break the vacuum.

The entire wafers which bonded with the optimal bonding parameters were diced to five areas according to the marks. Moreover, the chip units in each area were selected for scanning electron microscope (SEM) observation, thrust test and electrical performance test experiments.

### 3.1. SEM Test

**Figure 10:**
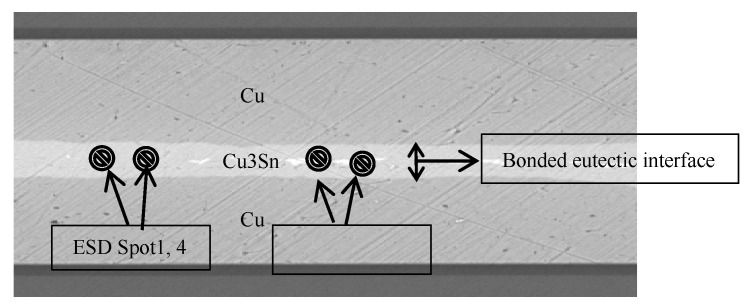
SEM photograph.

**Figure 11:**
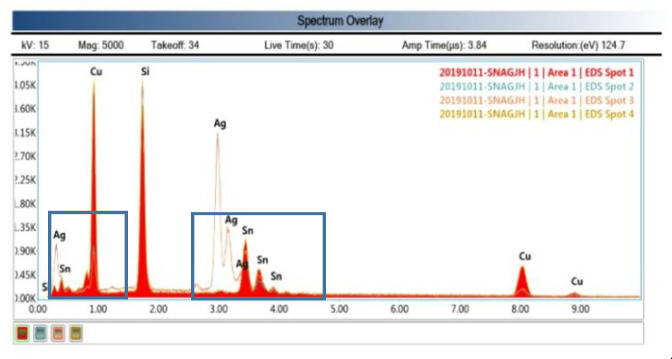
ESD spectrum.

Figure 10 and Figure 11 show the bonding interface obtained by using the parameters of Group 3 for pretreatment and bonding and the parameters of Group 4 for the wafer bonding. The bonding interface can be divided into three layers. The upper and lower layers are electroplated Cu layers. The middle is an inter-metallic compound (IMC) layer without via, cracks and cracks, which is formed by solid–liquid diffusion growth [22]. According to energy dispersive spectrometer (EDS) analysis, the ratio of the number of Cu atoms to the number of Sn atoms is about 11:3, and then the composition analysis of the cross sections of different bonding bumps is basically close to the atomic ratio of Cu_3_Sn, so the intermediate layer was determined to be Cu_3_Sn [23]. The Cu_3_Sn and Cu have similar density; when Cu_3_Sn grew on the surface of Cu, the change in volume was very small, so it could generate a dense structure, reasonable metal structure and bonding optimization conditions, there was not too much Cu_6_Sn_5_, avoiding the Kirkendall effect [24,25]. In addition, there were some white bright spots in the IMC layer, but they were not via. EDS analysis (spot2, 3) show they were mainly Ag. This was due to the electroplating process. The electroplated Sn layer was not pure Sn, there was a thin Ag layer existing, but it did not affect the IMC Layer formation [26,27]. The Si was detected in EDS analysis because the wafer was Si wafer and SiO_2_ on the surface. Si itself was an interference impurity. Only Cu and Sn play the role of eutectic.

### 3.2. Pushing Force Testing

**Figure 12:**
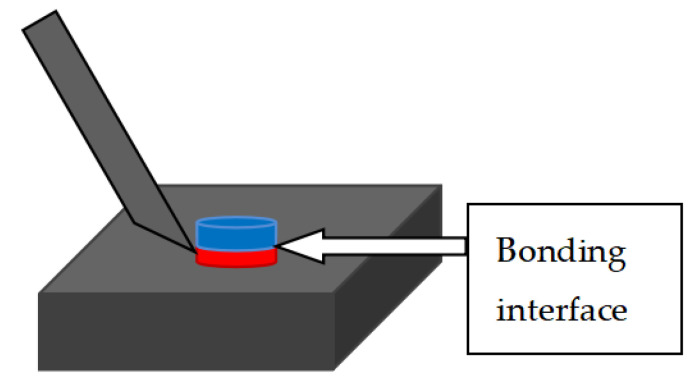
Schematic diagram of the pushing of the ball.

**Figure 13:**
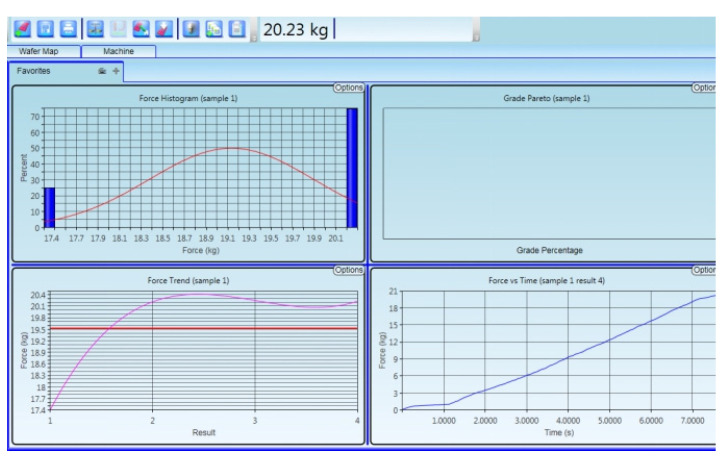
Result of the binding test after optimization of parameters.

To measure the reliability of semiconductor packaging machinery by pushing the crystal force, we used a ball-pushing machine to perform the ball pushing test from the bonding interface, tensile test speed is 700 μm/s, as shown in Figure 12. The test process was as follows: Choose a single die in different areas from the entire wafer and use a ball pusher to perform the bonding force test. This test does not need to remove the top wafer. It is found that the bonding strength of the entire large area is greater than 20.23 kg (unit area is 30.5 kg/cm^2^); the bonding strength is greater than the requirements of 18 kg/cm^2^. It shows in Figure 13 that the strength of the Cu–Sn bonding layer meets the requirements of the binding force test standard. The detection method is as follows: one of the five selected dies of one wafer is selected from the optimized wafers for thrust test [28].

### 3.3. Electrical Performance Test

**Figure 14:**
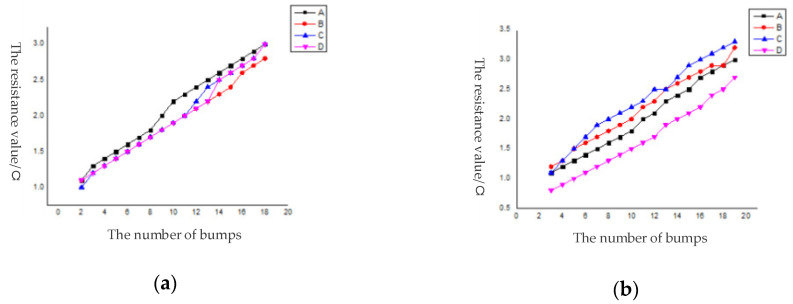

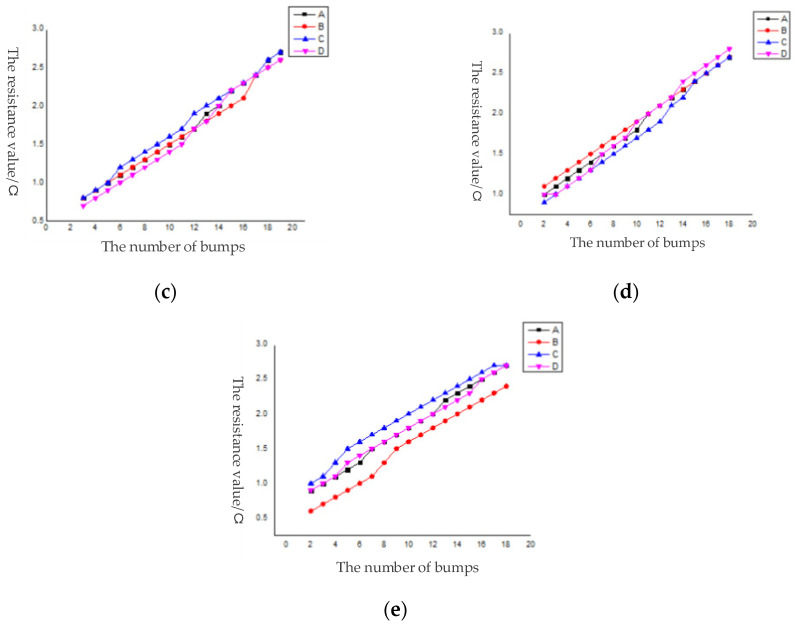
Resistance curve of daisy chain in each region. (**a**) die in Region 1; (**b**) die in Region 2; (**c**) die in Region 3; (**d**) die in Region 4; (**e**) die in Region 5.

**Figure 15:**
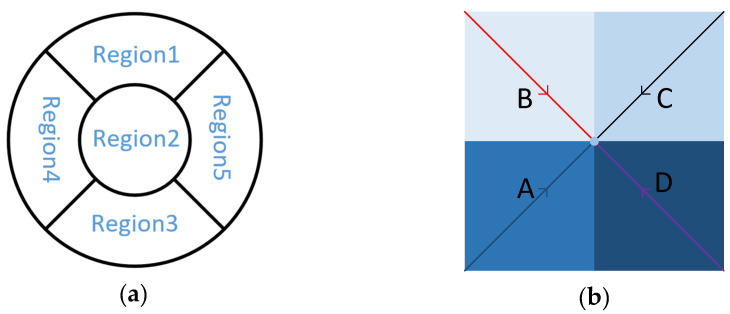
Schematic diagram. (**a**) Different regions on a wafer; (**b**) different serial circuits on a die.

**Figure 16:**
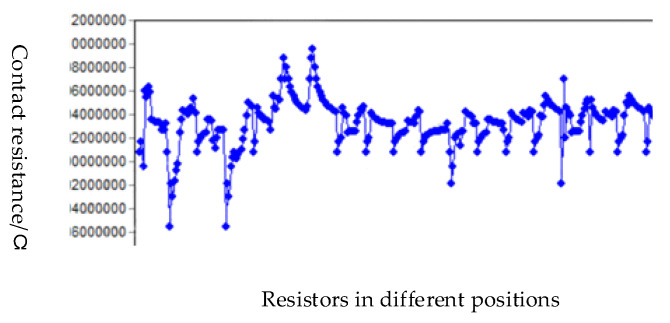
Statistics of contact resistance.

We conducted the circuit conduction test on the series bumps with daisy chain, added the DC signal to the pads at both ends of the daisy chain, used a multimeter to measure the series resistance and calculated the average value of the contact resistance as 3.35 mΩ [29]. There were 44,100 bumps in a single die. Among them, 3178 were distributed on the daisy chain. A total of 68 series routes were formed, all of which were conductive, and the conductivity was 100%. Among them, the bumps were activated on the daisy chain in each region. With the increase of the number of series bumps, the resistance value had a linear relationship with the number of bumps. Figure 14 was to select 5 dies in 5 regions of the wafer after bonding and calculate the resistance of each series line to obtain a graph of the number of bumps and the resistance value. Figure 15 was a schematic diagram of region selection on a wafer and a schematic diagram of serial circuits on a die. The distribution of the bumps in the four areas above a die was the same, according to the engineering drawing, the diagonal center is measured from the four corners. The contact resistance of each pair of bumps is shown in Figure 16.

In addition, we randomly selected 2 dies from each of the 5 regions of the bonded wafer for reliability experiments of high and low temperature impact 100 times (−65–150 °C) and high temperature storage 1000 h (150 + 10 °C). Performance test: After the test, the contact resistance was calculated to be 3.36 mΩ and 3.35 mΩ, respectively. All bumps distributed on the daisy chain in the tested die were turned on, and the conduction rate was 100%. It shows that bonding achieved good electrical performance and has high electrical and thermal reliability [10,30].

## 4. Conclusions

This paper studies a wafer-level, high-density and irreversible bump eutectic bonding technology. Based on the principle of Cu/Sn low-temperature bonding and the principle of testable current conduction, a bump bonding structure was designed, and a dense Cu_3_Sn IMC layer was obtained by optimizing bonding pretreatment and bonding process conditions. The bonding strength reaches and exceeds the industry standard. The current conductivity of the daisy chain was tested to be 100%. Reliability tests show that the performance of the CuSn solid–liquid diffusion IMC layer was stable. After the process of high temperature and high humidity, there was no reverse reaction of the compound at the bonding interface, and it had good thermal and mechanical reliability. The realization of wafer-level bonding of different size patterns showed that the wafer-level Cu/Sn bump bonding technology could meet the requirements of wafer-level TSV three-dimensional integration and multichip vertical interconnection.

## Figures and Tables

**Table 1:** Current density of different parts, brand of Cu and SnAg plating solutions.

Project	Current Density	Brand
RDL-Cu	1.726ASD	Chuangzhi Success Technology Co., Ltd.
UBM-Cu	2.5ASD	Chuangzhi Success Technology Co., Ltd.
UBM-SnAg	1.68ASD	Japan Ishihara Industry Co., Ltd.

**Table 2:** Parameters setting of different cleaning pretreatment before bonding.

Group	Plasma Cleaning	Citric Acid Cleaning	IPA Cleaning	Oven Baking
1	–	10 min	5 min	150 °C 10 min
2	Ar, N, H_2_ 5 min	10 min	5 min	150 °C 10 min
3	Ar, N_2_, H_2_ 5 min	10 min	5 min	–
4	Ar, N2, H2 5 min	Citric acid 5 min—quick dump rinse (QDR)—citric acid 5 min	5 min	–

**Table 3:** Parameter settings of different wafer-level bonding.

Group	Pretreatment	Prebonding	Bond	Cool Down
1	Raise temperature to 240 °C 2 min	–	280 °C (heating rate 10 °C/min) 7000 N 25 min	Cool down to 150 °C charge N_2_ to 80 °C
2	H_2_ reduction + raise temperature to 150 °C 2 min	–	280 °C (heating rate 10 °C/min) 7000 N 25 min	Cool down to 150 °C charge N_2_ to 80 °C
3	H_2_ reduction + raise temperature to 150 °C 2 min	Raise temperature and pressure to 240 °C (heating rate 15 °C /min) 7000 N 5 min	280 °C (heating rate 10 °C /min) 7000 N 25 min	Cool down to 150 °C charge N_2_ to 80 °C
4	H_2_ reduction + raise temperature to 150 °C 2 min	Raise temperature and pressure to 240 °C (heating rate 15 °C/min) 7000 N 5 min	280 °C (heating rate 10 °C/min) 7000 N 20 min	Cool down to 150 °C charge N_2_ to 80 °C
5	H_2_ reduction + raise temperature to 150 °C 2 min	Raise temperature and pressure to 240 °C (heating rate 15 °C/min) 7000 N 5 min	280 °C (heating rate 10 °C/min) 7000 N 15 min	Cool down to 150 °C charge N_2_ to 80 °C

## References

[1] Shih J.Y., Chen Y.C., Chiang C.H., Chiu C.H., Hu Y.C., Lo C.L., Chang C.C., Chen K.N. TSV-Based Quartz Crystal Resonator Using 3D Integration and Si Packaging Technologies. Proceedings of the IEEE Electronic Components & Technology Conference.

[2] Wu D.W., Li K.Z., Shan G.B., Wu L.S., Zheng X.Q., Jin Y.F. Opening the hole of the backside silicon and forming the alloy bump for TSVs. Proceedings of the 2015 16th International Conference on Electronic Packaging Technology (ICEPT).

[3] Li D., Shang Z., Yin S., Wen Z. (2017). Investigation of Au/Si Eutectic Wafer Bonding for MEMS Accelerometers. Micromachines.

[4] Ji W.U., Xie D.Q.J.M.E.T. (2014). Current status and trends of three-dimensional integrated technology. Mod. Electron. Technol..

[5] Ko C.T., Chen K.N., Lo W.C., Cheng C.A., Huang W.C., Hsiao Z.C., Fu H.C., Chen Y.H. Wafer-level 3D integration using hybrid bonding. Proceedings of the 3rd Systems Integration Conference.

[6] Chen S., Tanner D.M., Ramesham R., Ma H., Chen M., Xiong T., Liu S., Yi X. (2004). Wafer-level scale package of MEMS device by eutectic bonding method. Proc. Spie.

[7] Kecheng L.I., Liu X., Chen M. (2015). Developments of copper-to-copper low temperature bonding technology for 3D packaging. Electron. Compon. Mater..

[8] Garrou P., Bower C., Ramm P. (1971). Handbook of 3D Integration: Technology and Applications of 3D Integrated Circuits. Dig. Dis..

[9] Tang Y.-S., Chang Y.-J., Chen K.-N. (2012). Wafer-level Cu–Cu bonding technology. Microelectron. Reliab..

[10] Zuo Y., Shen J., Xu H., Gao R. (2017). Effect of different sizes of Cu nanoparticles on the shear strength of Cu-Cu joints. Mater. Lett..

[11] Yaping L. (2014). Research on Multilayer Stack Bonding Technology for System in Package.

[12] Nimura M., Mizuno J., Sakuma K., Shoji S. Solder/adhesive bonding using simple planarization technique for 3D integration. Proceedings of the 61st Electronic Components and Technology Conference (ECTC).

[13] Lapadatu A., Simonsen T.I., Kittilsland G., Stark B., Salomonsen G. (2010). Cu-Sn Wafer Level Bonding for Vacuum Encapsulation of Microbolometer Focal Plane Arrays. ECS Trans..

[14] Yoshihara S., Ohara F., Nagakubo M. (2003). Semiconductor device including eutectic bonding portion and method for manufacturing the same. U.S. Patent.

[15] Hoivik N., Wang K., Aasmundtveit K., Salomonsen G., Stark B. Fluxless wafer-level Cu-Sn bonding for micro- and nanosystems packaging. Proceedings of the 3rd Electronic System-Integration Technology Conference (ESTC).

[16] Cho Y.H., Kim S.E., Kim S. (2013). Wafer Level Bonding Technology for 3D Stacked IC. J. Microelectron. Packag. Soc..

[17] Ko C.T., Hsiao Z.C., Fu H.C., Chen K.N., Chen Y.H. Wafer-to-wafer hybrid bonding technology for 3D IC. Proceedings of the Electronic System-Integration Technology Conference.

[18] Mitchell J.S. (2008). Low Temperature Wafer Level Vacuum Packaging Using Au-Si Eutectic Bonding and Localized Heating. Ph.D. Thesis.

[19] Hetai L., Yechen L. (2002). Research of Silicon Bonding Technology. Sens. World.

[20] Fltgen C., Pawlak M., Pabo E., Weil H.J.V.D., Dragoi V. (2014). Wafer Bonding Using Cu-Sn Intermetallic Bonding Layers. Microsyst. Technol..

[21] Luu T.T., Duan A., Aasmundtvei K.E. (2013). Optimized Cu-Sn Wafer-Level Bonding Using Intermetallic Phase Characterization. J. Electron. Mater..

[22] Chang L.B., Yen C.I., You T.W., Jeng M.J., Wu C.T., Hu S.C., Kuo Y.K. (2014). Improving the reliability of eutectic bonding vertical power light-emitting diodes by a Mo buffer layer. Thin Solid Film..

[23] Liu H., Wang K., Aasmundtveit K.E., Hoivik N. (2012). Intermetallic Compound Formation Mechanisms for Cu-Sn Solid–Liquid Interdiffusion Bonding. J. Electron. Mater..

[24] Wang T., Shi J., Xie X. (1999). Study of die bonding technology for Au/In isothermal solidfication. J. Funct. Mater. Devices.

[25] He R., Fujino M., Akaike M., Sakai T., Sakuyama S., Suga T. (2017). Combined surface activated bonding using H-containing HCOOH vapor treatment for Cu/Adhesive hybrid bonding at below 200 °C. Appl. Surf. Sci..

[26] Park Y.S., Shin J.W., Choi Y.W., Paik K.W. A study on the intermetallic growth of fine-pitch Cu pillar/SnAg solder bump for 3D-TSV interconnection. Proceedings of the IEEE Electronic Components & Technology Conference.

[27] Shin J.W., Choi Y.W., Kim Y.S., Kang U.B., Paik K.W. Development of anhydride-based NCFs for Cu/Sn-Ag eutectic bonding and process optimization for fine pitch TSV chip stacking. Proceedings of the Electronic Components & Technology Conference.

[28] Mitchell J.S., Najafi K. A detailed study of yield and reliability for vacuum packages fabricated in a wafer-level Au-Si eutectic bonding process. Proceedings of the TRANSDUCERS 2009 International Solid-State Sensors, Actuators and Microsystems Conference.

[29] Zhang L., Jiao B., Ku W., Tseng L.-T., Kong Y., Chien Y.-H., Yun S., Chen D. (2017). An electrical test method for quality detecting of wafer level eutectic bonding. J. Micromech. Microeng..

[30] Gross D., Haag S., Reinold M., Schneider-Ramelow M., Lang K.-D. (2016). Heavy copper wire-bonding on silicon chips with aluminum-passivated Cu bond-pads. Microelectron. Eng..

